# Effect of hysterectomy on incidence trends of endometrial and cervical cancer in Finland 1953–2010

**DOI:** 10.1038/sj.bjc.6601763

**Published:** 2004-04-20

**Authors:** R Luoto, J Raitanen, E Pukkala, A Anttila

**Affiliations:** 1 Tampere School of Public Health, University of Tampere, FIN-33014, Finland; 2 Finnish Cancer Registry, Institute for Statistical and Epidemiological Cancer Research, Helsinki, Finland

**Keywords:** incidence, hysterectomy, age–period–cohort models, endometrial cancer, cervical cancer

## Abstract

The hysterectomy-corrected age-adjusted incidence rate of endometrial cancer was 29%, and for cervical cancer 11% higher than the uncorrected rate. Correction factors for such cancer sites are recommended for regular use. The levelling-off of the incidence of endometrial cancer appears to be an artefact caused by the increasing prevalence of hysterectomy.

Hysterectomy is one of the commonest surgical procedures for women with substantial regional and international variations in frequency (Carlson *et al*, 1993; Luoto *et al*, 1994; Settnes and Jorgensen, 1999). The proportion of women who have undergone a hysterectomy increases with age up to 55–59 years (van Keep *et al*, 1983; Luoto *et al*, 1994). The highest rates are in the United States, where approximately a third of women aged 45 years and over have undergone this operation. In Finland and in the United Kingdom, the corresponding fraction is at ages 45–64 years is approximately one-fifth (Walker and Jick, 1979; Vessey *et al*, 1992; Luoto *et al*, 1994; Clarke *et al*, 1995). In spite of reliable surveillance of hysterectomy rates, cancer rates have not been corrected for hysterectomy (Luoto *et al*, 1994).

We have used information on hysterectomy to derive uteri-at-risk and cervices-at-risk populations to correct gynaecological cancer rates in Finland.

## MATERIALS AND METHODS

To estimate the incidence of hysterectomy during the period 1950–1986, a table with 1-year calendar years as rows and 1-year age groups as columns, each cell including the number of cases and the population at risk, was formulated, from which birth cohort-specific rates could be derived.

The Finnish Hospital Discharge Register (FHDR) includes information on surgical procedures since 1987, but the codes were unreliable until 1990. Coding of hysterectomies was based on Nordic Version of ICD-9 coding of surgical procedures during the period 1987–1996, from 1997 on ICD-10 classification. For the present study, incidence rates for 5-year age groups and each year on 1991–1999 were calculated from FHDR.

The fit of the model-based prevalences was tested against prevalence rates of hysterectomy based on self-reports from three population-based health surveys in 1981, 1989 and 1997, with nearly identical questions on hysterectomy was nearly identical, including structural options on hysterectomy alone or with oophorectomy. First, the sample of Mini-Finland Health Study in 1981 included 8000 persons 30–99 years of age, half of which were women. Almost 90% of them underwent both health interview and clinical examination (Aromaa *et al*, 1989). In 1989, prevalence of hysterectomy was obtained from a mail questionnaire survey among 45- to 64-year-old Finnish women (*N*=2000) randomly sampled from the Population Register. The response rate was 86% (Luoto *et al*, 1994). Third point prevalence of hysterectomy was based on the FINRISK mail questionnaire survey in 1997, covering a random sample of population in five regions: two in eastern, one in southwestern and one in Northern Finland together with one in the Helsinki capital area (Vartiainen *et al*, 1998). Response rate was 75% among the 3763 of female respondents aged 25–64 years.

Corrected estimates of endometrial, cervical and ovarian cancer incidence were produced by multiplying the cancer incidence rates in 5-year age groups by a correction factor (the percentage of hysterectomised women). The Poisson's model including interaction between cohort and period was extrapolated for the years 2000–2010 to obtain future rates.

## RESULTS

### Incidence and cumulative incidence of hysterectomies

From 1991 to 1999, the annual number of hysterectomies (11 956 in 1999) increased by 16% (Table 1

**Table 1:** Number (*N*) and incidence of total and partial hysterectomies in Finland 1991–1999, and the incidence of endometrial and cervical cancer with and without correction for hysterectomies

	**Incidence of total hysterectomy**		**Incidence of endometrial cancer/10^6^**		**Incidence of partial hysterectomy^a^**		**Incidence of cervical cancer/10^6^**	
**Year**	***N***	**/10^6^**	**Uncorrected**	**Corrected**	***N***	**/10^6^**	**Uncorrected**	**Corrected**
1991	10 333	3162	134	158	8237	2500	28	30
1992	10 619	3185	137	162	8398	2497	33	35
1993	10 571	3089	142	171	8733	2535	37	39
1994	10 554	3022	136	165	8890	2531	37	40
1995	10 169	2855	135	166	8900	2487	44	48
1996	10 759	2981	153	190	9923	2741	44	47
1997	11 816	3247	142	179	10 935	3001	39	42
1998	12 227	3315	159	203	11 351	3071	45	49
1999	11 956	3208	146	188	11 246	3017	38	42

**Figure 1:**
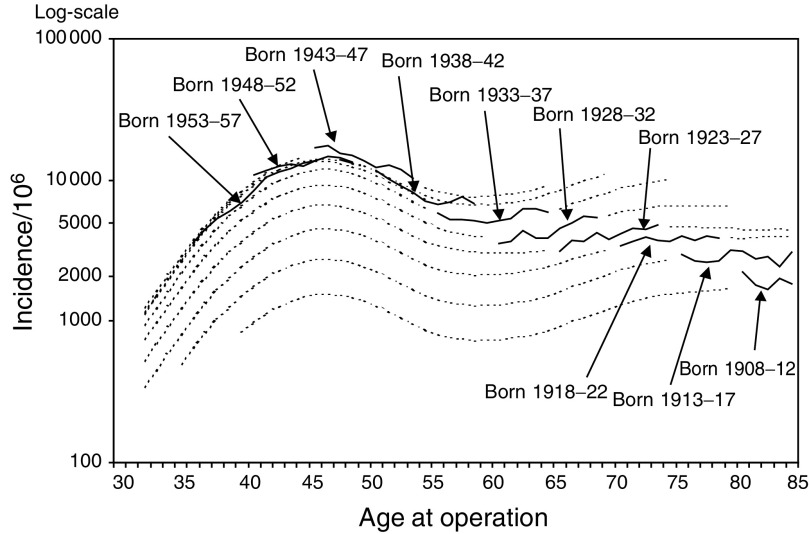
Incidence of hysterectomy by age and birth cohort, 1987–1999 based on Finnish Hospital Discharge Register. Logarithmic scale.

All incidence rates are adjusted for age to the world standard population.

a Number of women with hysterectomy, cervix intact.

). The incidence of partial hysterectomies (hysterectomies with cervix intact) increased from 2500 in 1991 to 3017 per million women in 1999. The age-specific incidence of hysterectomy increased until the 1990s, but changed in the 1990s to a decrease among the fertile aged women (age <50 years). In the birth cohort-specific hysterectomy rates, an increase up to the birth cohort born 1943–1947 was observed, after which the incidence of hysterectomy started to decrease (Figure 1

). The first peak age at hysterectomy was observed around 47 years and the second in the age group 70–80 years.

**Figure 2:**
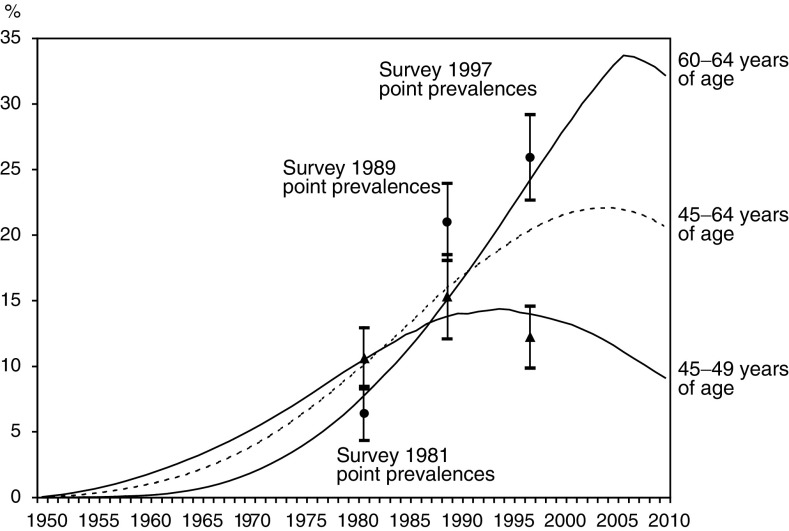
Age-specific prevalence rates of hysterectomy in 1950–2010 in age groups 45–49 years (black line) and 60–64 years (grey line), based on estimated incidence rates. The vertical bars indicate the respective point prevalence rates of the three population-based surveys (Aromaa *et al*, 1989, Luoto *et al*, 1994, Vartiainen *et al*, 1998) together with their 95% confidence intervals. Dotted line shows the time trend of age-adjusted prevalence of hysterectomy in age range of 45–64 years.

Cumulative incidence rates (later referred to as ‘prevalence’) of hysterectomy in the age group 45–49 years have decreased since 1989 (Figure 2

). The prevalence of hysterectomy continues to increase in age group 45–64 years of age, and even more steeply among older women of age 60–64 years of age. However, even in the oldest group this decreases after the year 2007. The estimated prevalence was mostly parallel to earlier published rates except in 1989 for the age group 60–64 years (Table 2

**Table 2:** Prevalence of hysterectomy among women aged 45–64 years in Finland in population-based health surveys 1981, 1989 and 1997

	**Age group**				
**Survey**	**45–49 years**	**50–54 years**	**55–59 years**	**60–64 years**	**Total**
1981^a^					
Hysterectomised	47	46	27	23	143
Prevalence (%)	10.6	10.9	8.4	6.4	9.3*
					
1989^b^					
Hysterectomised	49	89	72	104	314
Prevalence (%)	15.3	20.4	18.2	21.0	18.5*
					
1997^c^					
Hysterectomised	60	102	122	120	404
Prevalence (%)	12.2	21.5	23.7	25.9	20.0*

a Aromaa *et al* (1989).

b Luoto *et al* (1994).

c Vartiainen *et al* (1998).

* Adjusted for age to the world standard population.

).

### Cancer incidence

**Figure 3:**
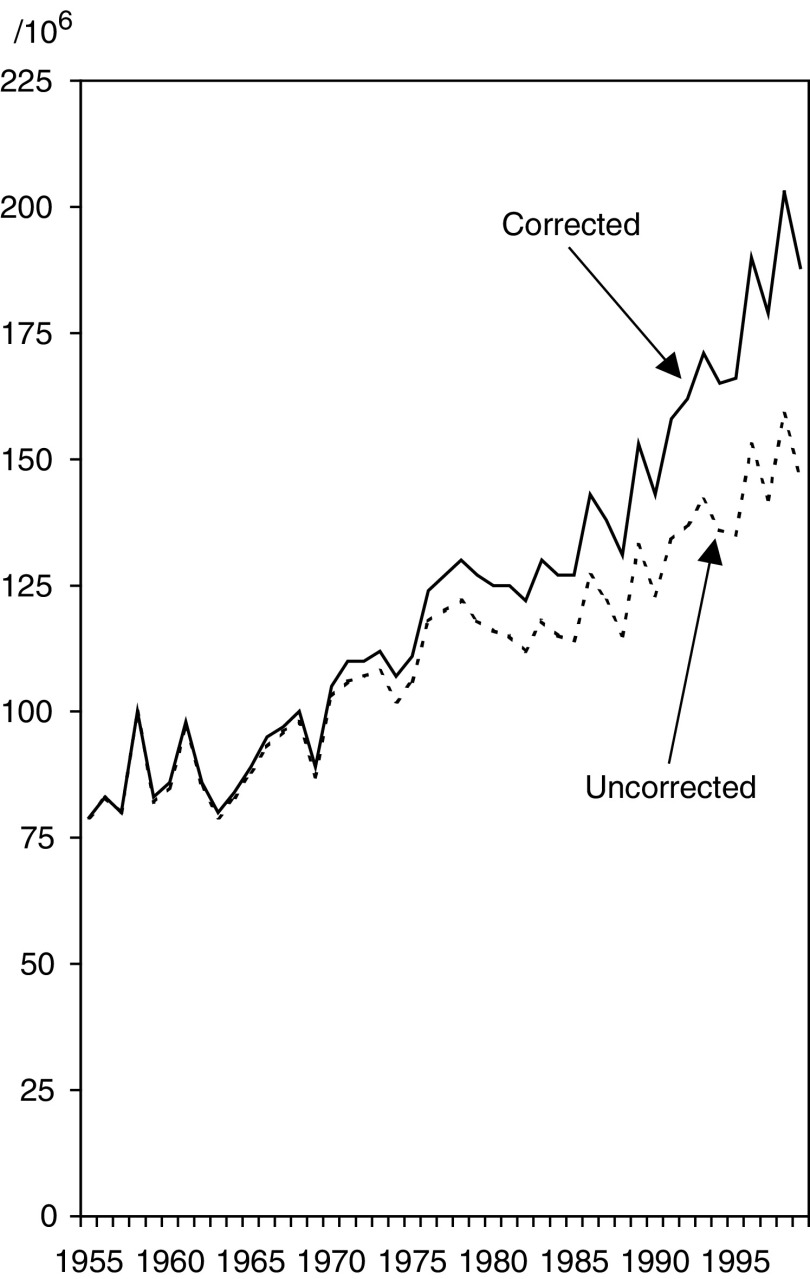
Age-adjusted incidence of endometrial cancer in Finland 1953–1999 – uncorrected and corrected for prevalence of hysterectomy.

The age-adjusted incidence of endometrial cancer in 1999 was 146/10^6^, but 188 per million after applying uteri-at-risk corrections (Table 1). Uncorrected endometrial cancer rates show a levelling-off in the 1980s, which is less apparent in corrected rates (Figure 3

).

In the age group 50–59 years, the difference between uncorrected and corrected rate of endometrial cancer is large and up to 32% (Table 3

**Table 3:** Uteri-at-risk-corrected and -uncorrected age-adjusted incidence rates of endometrial and cervical cancer in Finland 1995–1999.

	**Endometrial cancer/10^6^**					**Cervical cancer/10^6^**				
	<**40 years**	**40–49 years**	**50–59 years**	**60–69 years**	**70+ years**	<**40 years**	**40–49 years**	**50–59 years**	**60–69 years**	**70+ years**
Uncorrected	3.5	116	429	874	789	19.6	88.4	64.8	99.1	130.7
Corrected	3.5	131	566	1134	912	19.7	95.2	76.4	114.7	145.5
Increase (%)	1.0	12.9	32.0	29.7	15.7	0.4	7.7	17.8	15.7	11.3

). The impact of correction is smaller in cervical cancer, but even here the corrected rate of cervical cancer differs by up to 17.8%. The corrected age-adjusted trend in the incidence of cervical cancer was almost unchanged as compared to the uncorrected trend (Figure 4

**Figure 4:**
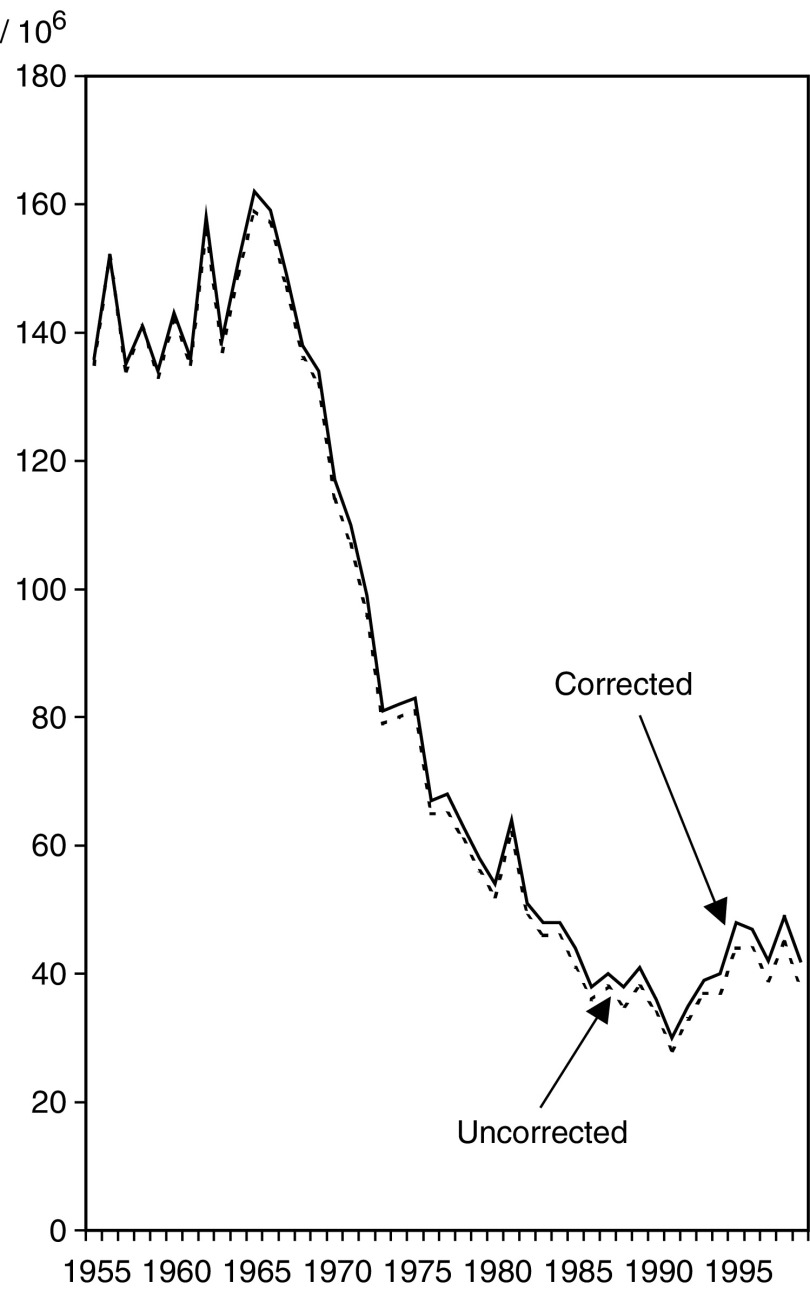
Age-adjusted incidence of cervical cancer in Finland 1953–1999 – cervix-at risk-corrected and -uncorrected trends for prevalence of having cervix removed.

).

## DISCUSSION

When the corrected population at risk was used rather than the entire population, the incidence of endometrial cancer was 29%, cervical cancer 11% and ovarian cancer 8% higher. Uncorrected endometrial cancer rates showed a levelling-off in the 1980s not seen in corrected rates, explained by the increasing prevalence of hysterectomy a change not recognised previously. Such correction has not been systematically used in any country, although the first recommendations for correction were published 24 years ago (Lyon and Gardner, 1977) and the topic is still discussed (Merrill *et al*, 2001).

Our results were in keeping with the first US studies in the 1970s, showing a 20–45% increase in endometrial cancer incidence rate depending on the age group and calendar year (Lyon and Gardner, 1977; Merrill and Feuer, 1996) applied a risk-adjusted incidence method for cervical cancer and found that in the period 1990–1992 the age-adjusted incidence rate increased by 15%. The greatest increase, by 58%, was seen among women aged 65–69 years. Correction for the scale of hysterectomies in Canada in 1964–1966 to 1974–1966 converted an apparent fall in mortality from cancer of the endometrium to stability (Miller *et al*, 1981).

Our estimates of hysterectomy prevalence were based on reliable data on hysterectomy incidences over a 12-year period, obtained from a population-based hospital discharge register. The incidence trends were also extrapolated backwards to obtain estimates over the preceding life of the women. The validity of these extrapolations was confirmed by comparing the cumulative incidence rates with age-specific prevalence rates available from three cross-sectional population surveys.

The small surveys samples (especially that in 1989) made the point prevalences of hysterectomy relatively imprecise. However, our estimates fall within their intervals except for that in 1989 for the age group 60–64 years, with estimated prevalence markedly lower than that found. On the other hand, our estimated cumulative incidence rates were consistent with the two other survey results for this age group.

Coverage of FHDR is at least 90% compared to hospital records (Keskimäki and Aro, 1991). Generally, benefits of register information compared with cross-sectional surveys are clear, although cross-sectional hysterectomy results combined with mortality data analysed using a life-table method can produce similar results (Merrill, 2001, Merrill *et al*, 2001).

The incidence of hysterectomy is higher in Finland than in other Nordic countries (Luoto *et al*, 1994, Vuorma *et al*, 1998) is possibly related both to its health-care system with private gynaecologists and the increasing use of hormonal replacement therapy. Every third woman in Finland is currently using hormonal replacement therapy (Topo *et al*, 1999), which is known to enhance uterine fibroid proliferation, the most frequent indication for hysterectomy.

Indications for, and time trends of, hysterectomy vary in different countries, although differences are not large. Correction factors which therefore need to be country specific and stratified by age and time have been developed from 1998 onwards (Giersiepen *et al*, 2003). In those European countries where neither hospital-based data nor reproductive health surveys are available, a special survey is needed to obtain correction factors. Historical data on hysterectomy rates, required for correcting incidence trends, may be available only from register-based information sources as used in this study. In Finland, the correction has a material effect only on the incidence of endometrial cancer: the levelling-off in the 1980s of the incidence seems not to be real but an artefact caused by the increasing prevalence of hysterectomies.

A recent comparison of hospital discharge data and cross-sectional survey for correcting uterine cancer incidence (Merrill *et al*, 2001) concluded that both methods may provide correct estimated of burden of gynaecological cancer. The results of the present study confirm this view.
