# Effect of hysterectomy on incidence trends of endometrial and cervical cancer in Finland 1953–2010

**DOI:** 10.1038/sj.bjc.6602288

**Published:** 2004-11-23

**Authors:** R Luoto, J Raitanen, E Pukkala, A Anttila

**Correction to:** *British Journal of Cancer* (2004) **90**, 1756–1759. doi:10.1038/sj.bjc.6601763

Due to an error, Table 1

**Table 1:** Number (*N*) and incidence of total and partial hysterectomies in Finland 1991–99, and incidence of endometrial and cervical cancer with and without correction for hysterectomies

	**Overall incidence of hysterectomy^a^**		**Incidence of endometrial cancer/10^6^**		**Incidence of total hysterectomy^b^**		**Incidence of cervical cancer/10^6^**	
**Year**	***N***	**/10^6^**	**Uncorrected**	**Corrected**	***N***	**/10^6^**	**Uncorrected**	**Corrected**
1991	10 333	3162	134	158	8237	2500	28	30
1992	10 619	3185	137	162	8398	2497	33	35
1993	10 571	3089	142	171	8733	2535	37	39
1994	10 554	3022	136	165	8890	2531	37	40
1995	10 169	2855	135	166	8900	2487	44	48
1996	10 759	2981	153	190	9923	2741	44	47
1997	11 816	3247	142	179	10 935	3001	39	42
1998	12 227	3315	159	203	11 351	3071	45	49
1999	11 956	3208	146	188	11 246	3017	38	42

a All incidence rates are adjusted for age to the world standard population.

b Number of women with hysterectomy, cervix removed.

and some text on page 1756 of the above paper were shown incorrectly. The correct Table 1 and text are given below:

On page 1756 of this article, the second sentence in the Results section should read as follows:

The incidence of hysterectomies with cervix removed increased from 2500 in 1991 to 2017 per million women in 1999.

