# Supplementary material for: Effect of hysterectomy on incidence trends of endometrial and cervical cancer in Finland 1953–2010
Source: Br J Cancer. 2004 Apr 20;90(9):1756–9. doi: 10.1038/sj.bjc.6601763 (PMC2409756; doi:10.1038/sj.bjc.6601763)
Supplement: Supplementary Information [file 90-6601763x1.pdf]

Methods: supplementary online material

We assumed that the observed annual numbers of hysterectomies y_{ap}, where *a* refers to age and *p* to period, are Poisson distributed variables with the expected value λ_{ap}. The logarithm of the hysterectomy incidence rate was modelled as

$$\log\left(\frac{\lambda_{ap}}{N_{ap}}\right) = \mu + \alpha_a + \beta \cdot p + \gamma_c + \gamma_{c*} . \beta_{p*} ,$$

where *N* represents person-years in age-group *a* and period *p*, μ is constant, α_a represents the effect of age-group *a*, $\beta \cdot p$ the effect of period drift and γ_c the effect of birth cohort *c*. The term $\gamma_{c*} \cdot \beta_{p*}$ represents interaction between cohort and period, which was included after year 1985.

This model was extrapolated for years 1950-1990 and 2000-2007 in order to get incidence estimates before and after the FHDR coverage period.

Prevalence was calculated by cumulating the incidences based on the above described modelling. Usually population at risk in cancer incidence calculations include also hysterectomized women, i.e., women without uterus or ovary. Therefore population at risk has to be corrected by e^{-CI} (CI=cumulative incidence), when the prevalence is based on the formula

$$prevalence_y = prevalence_{y-1} + \frac{x_{yi}}{N_{yi} e^{-prevalence_{y-1}}} ,$$

where x_{yi} is estimated number of cases in the year y in age-group i and N female population during the same year in the same age-group. GLIM program has been used in estimating the parameters.
